# Prevalence and Determinants of Self-Medication Practices among Cardiovascular Patients from Béja, North West Tunisia: A Community-Pharmacy-Based Survey

**DOI:** 10.3390/pharmacy12020068

**Published:** 2024-04-12

**Authors:** Maria Suciu, Lavinia Vlaia, Eya Boujneh, Liana Suciu, Valentina Oana Buda, Narcisa Jianu, Vicențiu Vlaia, Carmen Cristescu

**Affiliations:** 1 Department II—Pharmacology-Pharmacotherapy, “Victor Babeș” University of Medicine and Pharmacy, Eftimie Murgu Sq. No. 2, 300041 Timișoara, Romania; suciu.maria@umft.ro (M.S.); suciu.liana@umft.ro (L.S.); carmencristescu@umft.ro (C.C.); 2 Research Center for Pharmaco-Toxicological Evaluation, “Victor Babeș” University of Medicine and Pharmacy, Eftimie Murgu Sq. No. 2, 300041 Timișoara, Romania; narcisa.dinu@umft.ro; 3 Department II—Pharmaceutical Technology, Formulation and Technology of Drugs Research Center, “Victor Babeș” University of Medicine and Pharmacy, Eftimie Murgu Sq. No. 2, 300041 Timișoara, Romania; vlaia.lavinia@umft.ro; 4 Tunisian Pharmacist, Abdellatif Boujnah Pharmacy, Avenue Mongi Slim, Béja 9000, Tunisia; eyaboujneh2@gmail.com; 5 Department I—Clinical Pharmacy, Communication in Pharmacy, Pharmaceutical Care, “Victor Babeș” University of Medicine and Pharmacy, Eftimie Murgu Sq. No. 2, 300041 Timișoara, Romania; 6 Organic Chemistry, Formulation and Technology of Drugs Research Center, “Victor Babeș” University of Medicine and Pharmacy, Eftimie Murgu Sq. No. 2, 300041 Timișoara, Romania; vlaiav@umft.ro

**Keywords:** self-medication, cardiovascular patients, prevalence, healthcare practitioners, Tunisia

## Abstract

In Tunisia, self-medication is a common practice, and there is a continual rise in the prevalence of cardiovascular disease. Given the lack of data on the self-medication practices (SMPs) among cardiovascular patients in this area, the present study aimed to identify the prevalence and determinants of SMPs among cardiovascular patients in the city of Béja. A community-pharmacy-based survey was conducted among selected cardiovascular patients in Béja, Tunisia, from May 2021 to June 2021. Data were collected using a self-administered questionnaire provided by pharmacists during in-person surveys with patients. Descriptive statistics were used to summarize the data, while Fisher’s exact test was used for categorical variables, with the significance level set at *p* < 0.05. The frequency of self-medication among the 150 respondents was 96%; 70.14% of participants reported that the primary reason why people engage in self-medication is the existence of an old prescription. The most prevalent conditions leading patients to self-medicate were headaches (100%), fever (83.33%), toothache (65.97%), and dry cough (47.92%). The most frequently self-administered drugs were paracetamol (100%), antibiotics (56.94%), and antitussives (47.92%). The results of our study indicate that SMPs among Tunisian cardiovascular patients have a high prevalence. With this in mind, healthcare practitioners should ask their patients about their self-medication practices and advise cardiovascular patients about the risks and benefits associated with this practice.

## 1. Introduction

The recent transition experienced by Tunisia on various levels, including economic, social, and political, is also reflected in population health, as evidenced by an increase in the incidence of cardiovascular disease (CVD) [1]. Ischemic heart disease and stroke are the two leading causes of premature death worldwide [2], including in Tunisia, according to statistical data from 2019 [3]. To date, there are no representative data on the prevalence of CVDs in the adult population of Tunisia. According to recent data, only the prevalence of hypertension in the population has been analyzed, showing an upward trend in recent years. Recent studies have focused only on the prevalence of hypertension in the population, with their results indicating an increasing trend in this condition, from 28.9% in 1995 [4] to 38.1% in 2019 [5]. A more recent study by Jemaa et al. [6] pointed out a further increase in hypertension prevalence to 50.5% in 2020.

According to the World Health Organization (WHO), self-medication practice (SMP) is defined as the choice and use of medicines by an individual, on their own initiative, without the advice of a health professional, to improve/treat symptoms or self-diagnosed clinical situations [7]. SMPs also include the intermittent or continuous use of a medicine by the patient, according to a diagnosis and a medical prescription previously received from the prescriber for the treatment of a recurrent/chronic disease. It also involves the use of family members’ medications or the administration of drugs based on the advice of family and/or friends [7]. The decision to choose and utilize medication is entrenched in the patient’s own knowledge and expertise, without seeking specialized advice (medical or pharmaceutical) [7].

SMP is a main component of the self-care process, which involves a healthy lifestyle and the recognition of symptoms and their severity, but also the responsible use of medicines and self-monitoring of a disease’s evolution [7].

Reducing the burden of diseases and improving the functional, physical, and mental status, as well as health-related quality of life, in patients are important components of the healthcare of all patients, including those with CVDs. In these conditions, resorting to SMP is regarded as a quick and easy solution for everyone, including patients and healthcare professionals, to reduce the burden of both acute and chronic diseases, whether acute or chronic. This approach may offer economic and public health benefits, but it also carries the risk of bypassing good practice and/or engaging in the misuse of medicine [8,9,10,11].

For the patient, SMP offers many benefits, including the rapid improvement of their general condition, lessening the emotional impact of a medical consultation, boosting confidence in their ability to manage a medical situation, and saving time and money. However, the risks arising from the irresponsible use of medicines are also present and worrying. These risks increase with the age of the patient, the presence of various comorbidities (cardiovascular, metabolic, digestive, etc.), and the concomitant use of other drugs, which can generate interactions and potentially lead to severe adverse events [12]. At the community level, exposing patients to irrational use of medicines can trigger an increase in the incidence of drug-induced diseases and health insurance costs. The therapeutic management of the cardiovascular (CV) patient may also be compromised by the inappropriate use of drugs in SMPs [13,14].

Despite the large share of SMPs in the population of different countries and their potential negative impact on the healthcare status, studies on their prevalence and associated effects on high-risk population groups (children, the elderly, CV patients, diabetics, etc.) are limited [15,16]. To the best of our knowledge, only a few studies published in the last two decades have evaluated SMPs among CV patients. According to a recent review [17], SMPs are found in over 70% of hypertensive patients across 18 countries around the world, and Veliz-Rojas et al. suggested that the prevalence of SMP in Chile is as high as 98.7% among patients enrolled in a primary cardiovascular healthcare program [18]. According to the results of a few studies, the prevalence of SMPs in Tunisia are high in both the pediatric population (especially with antibiotics) [19,20] and general population (56%) [21].

From the perspective of the current and future economic situation, given that the global healthcare system will undergo significant changes in the coming years, self-care and self-medication practices will play an increasingly important role in the context of new changes, imposing themselves as ways to reduce these costs [8].

Therefore, it is crucial to investigate the self-medication practices among cardiovascular patients in Tunisia, as this population may be at higher risk due to the lack of representative data on the prevalence of cardiovascular diseases and the increasing incidence of hypertension. Understanding the prevalence and determinants of self-medication practices can provide insights into the healthcare-seeking behavior of cardiovascular patients in Tunisia. Moreover, identifying the factors that influence self-medication practices can help healthcare professionals develop targeted interventions to promote safe and appropriate medication use among this population.

To address this knowledge gap, a study was conducted in Béja to investigate the self-medication practices among cardiovascular patients. The study aimed to (1) evaluate the prevalence and determinants associated with SMPs in the selected population and (2) identify a profile of the Tunisian CV patient predisposed to practice self-medication.

## 2. Materials and Methods

### 2.1. Study Design, Area, and Period

This non-interventional cross-sectional study employed surveys over a two-month period (between 1 May 2021, and 30 June 2021) in two independent pharmacies in Tunisia, in Béja. Béja is a town in northern Tunisia, the capital of the Béja Governorate, with a population of over 100,000 inhabitants, as reported in 2014 [22,23]. There are 13 day pharmacies and 2 night pharmacies in Béja. The two independent pharmacies (involved in the present study) were day pharmacies, situated in the northern part of the city, near a regional hospital. The study was based on real-life practice data and was performed as an in-person survey among the patients who visited the two community pharmacies. A pilot test was conducted before performing the survey in one of the pharmacies, in order to assess the readability, reliability, and validity of the questionnaire (i.e., improve its comprehensibility, correct its flaws, and delete unnecessary questions in order to reduce the time needed to complete it). More precisely, the questionnaire was given to six patients in one of the pharmacies on two occasions. Then, in order to evaluate the constancy of the questions, a reliability scale evaluation was performed, leading to a good Cronbach’s alpha score of 0.815. The data collected from the pilot test were not included in the final results. Moreover, the final version of the questionnaire (Form S1) was obtained after consulting a psychologist.

### 2.2. Source of Study Patients

All patients, customers of the two independent pharmacies, who self-reported that they are registered with a CVD or regularly administer CV medication, were considered the source population of the study. The participants were recruited based on simple random sampling.

### 2.3. Inclusion and Exclusion Criteria

- Inclusion criteria

All adults (over 18 years old) who visited one of the two pharmacies during the two months, live in the surveyed community, possess a basic level of reading comprehension and the ability to understand French text, self-reported that they are on medical records or undergoing treatment for at least one CV condition, and agreed to respond to the questionnaire were included in this study.

- Exclusion criteria

Patients who previously participated in another similar survey, had language barriers (inability to communicate in French) or obvious signs of cognitive impairment, or expressed their refusal to participate were not included in the study.

### 2.4. Data Collection Procedure and Tool

The pharmacists employed by the participating pharmacies collected the data using a self-administered questionnaire for all the patients who self-reported at least one CVD. The questionnaires were completed by pharmacists during a face-to-face survey with patients. They were invited to participate in the survey on a voluntarily basis, and those who agreed to participate were assured of the confidentiality of the discussion and the data provided.

- The investigators

For data collection, two pharmacists (one from each pharmacy) with good communication skills in local languages (French and Arabic) and experience in the practice of the profession were recruited. Each patient was informed about the purpose and structure of the questionnaire (clarifying any ambiguity/misunderstanding). The questionnaire was structured to allow the collection of necessary information regarding the socio-demographic characteristics and lifestyle habits of CV patients, the prevalence and determinants of SMPs being among of them.

- Study tool: the questionnaire

The questionnaire consisted of five sections. The first section contained questions regarding socio-demographic information, including age, sex, marital status, level of education, area of residence, employment status, and socio-economic level (self-reported by the patient). Information about their lifestyle habits, presence/absence of CVD, and whether they have engaged in SMPs in the past 6 months was collected in the second section. The third section was used to assess the reasons for SMP, source of advice, source of drug acquisition, outcomes obtained from drug administration, and other information regarding SMPs. The fourth section focused on the most common symptoms/diseases for SMPs, as well as the most common therapeutic classes of drugs used for SMPs. The last section of the questionnaire was used to assess all associated comorbidities and concurrent chronic medications taken by the patient (the medical and medication history of each patient).

Regarding the marital status variable, it should be mentioned that the respondents were analyzed on separate categories, namely cohabitation with a partner, divorced, widowed, and single (which included those persons who were not involved in a marriage or were not in a relationship). The education level of the patients was assessed according to the International Standard Classification of Diplomas (ISCED 2011) [24] and was classified as low, moderate, or high.

The questionnaire was written in French. The questions were formulated to avoid influencing patients’ answers and were either open or closed, with a single answer or multiple-choice answers. The purpose of the study and a brief definition of SMPs were provided to patients, both verbally by the investigating pharmacist and in writing in the questionnaire form.

### 2.5. Variables Studied

- Independent variables: age, marital status, educational level, residence, employment status, socio-economic level, smoker status, alcohol intake, dietary practices, and physical activity.
- Dependent variable: self-medication practice (yes or no).

### 2.6. Operational Definition

In our study, self-medication has been defined as the self-reporting by study participants of the consumption of at least one medicine or supplement, on their own initiative, in the last six months (the timeframe we have chosen) for the treatment of self-recognized diseases or symptoms, without medical/pharmaceutical supervision or intervention. This may include using previously prescribed medications in similar situations.

SMP refers to the act of a study participant using a drug in the last 6 months as a result of the suspicion of a disease or the recurrence of previously known symptoms and without a current prescription from a physician.

### 2.7. Statistical Analyses

Windows version 17.0 of the Statistical Science Package (SPSS) (IBM, Armonk, NY, USA) was used for processing coded data extracted from questionnaires and analyzing the relationship between different variables. The free trial version of MedCalc statistical software (version 22.023) was also used [25]. When describing the sample population, two types of variables were calculated: continuous variables, expressed as means ± standard deviations (SD), and qualitative variables, described using frequency and percentages. Descriptive statistics were used to analyze the relationships between the different variables studied; data are presented as odds ratios (ORs) with 95% confidence intervals (Cis). The Shapiro–Wilk test was used to evaluate the type of data distribution, revealing a normal distribution of the data. The association between socio-demographic variables and lifestyle habits and whether or not Tunisian CV respondents engaged in SMP was evaluated using Fisher’s exact test. *p* < 0.05 was considered to indicate statistical significance in the analysis of the differences between the patient groups in the study.

The determinants of SMPs were analyzed for two groups of CV patients: those who practiced self-medication (SMP—yes) at least once in the last six months and those who did not practice self-medication (SMP—no).

### 2.8. Ethical Considerations

All participants who agreed to take part in this survey and were found to be eligible were informed of the objectives of the study and provided verbal informed consent prior to a face-to-face survey with the investigating pharmacist. The self-administered questionnaires were numbered in the order of enrolment of the respondents in the study and did not contain identifying data of the interviewees. Participants’ sensitive data and information, such as their names, addresses, and other personal contact details, were not collected. Thus, all the information obtained after completing the questionnaires was stored in an irreversibly anonymized fashion.

In accordance with the current legislation of Tunisia [26], the ethics agreement and rules of good practice of studies apply only in the case of interventional research. As the present survey referred to an observational, non-interventional study, no ethical approval was required. However, the opinion of the Conseil National de l‘Ordre des Pharmaciens de Tunisie was sought, which referred to the above-mentioned Tunisian regulation specifying that the agreement of the two pharmacies is required to conduct these surveys, without mentioning their names. Consequently, the participating pharmacy agreements for our research group were obtained before starting the study.

Finally, our research was conducted in accordance with the Declaration of Helsinki.

## 3. Results

### 3.1. Prevalence of Self-Medication among Tunisian CV Patients

The current survey included a total of 160 Tunisian CV patients who visited the participating pharmacies during the study period. Of these, 150 (93.75%) of them agreed to participate in our survey and were included in the analysis group.

A total of 144 (96%) of the surveyed Tunisian CV patients reported using medicines without medical advice or a specialized medical recommendation, for various reasons.

### 3.2. Socio-Demographic Characteristics and Lifestyle Habits of Study Participants

A total of 64% of respondents were men, and 86.67% of participants lived alone (following a divorce, death of a partner, etc.). A total of 33.33% of the participants were between 20 and 40 years old, and the mean age was 43.65 years (standard deviation of ±1.29 years). The majority (70%) of the surveyed patients came from urban areas. Half of the total study participants had a moderate educational level, and 51.34% had a low economic status. In total, 24% of the participants were self-employed, and over 30% were retired.

According to the self-reported lifestyle habits of Tunisian CV patients, most respondents were smokers (75.33%), 58.67% did not practice any physical activity, 63.33% of the study participants did not consume alcohol, and 57.33% practiced a low-sodium diet (Table 1).

### 3.3. Cardiovascular History Profile of Tunisian Patients Who Practiced Self-Medication

**Figure 1:**
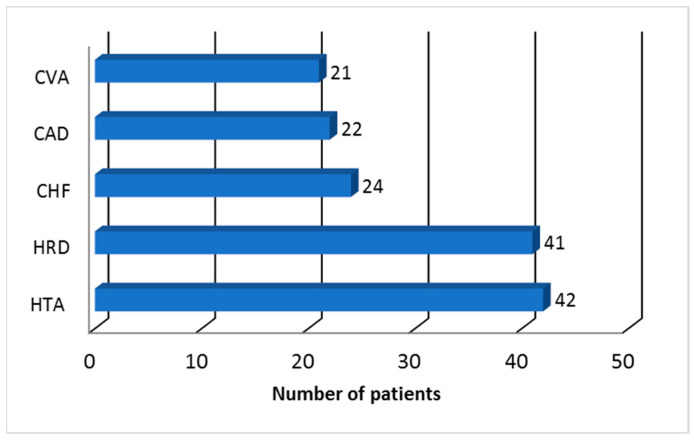
Cardiovascular history profile of Tunisian patients who practiced self-medication (CAD: coronary artery disease; CHF: congestive heart failure; CVA: cerebrovascular accident; HRD: heart rhythm disorders; HTA: arterial hypertension).

The analysis of the patients’ cardiovascular history highlighted the presence of the top three diseases that affect the cardiovascular system, as follows: essential arterial hypertension in 42 (28%) respondents, heart rhythm disorders (such as atrial fibrillation or flutter) in 41 (27.33%) respondents, and chronic heart failure in 24 (16%) respondents (Figure 1).

### 3.4. Reasons for Engaging in Self-Medication

Table 2 shows significant differences in the personal reasons for SMPs among the Tunisian CV patients included in the study.

The main reasons for self-medication were reported to be the possession of a previous doctor’s prescription (101, 70.14% of respondents) and/or because of time and money savings (62, 43.06% of respondents and 52, 36.11% of respondents, respectively).

**Figure 2:**
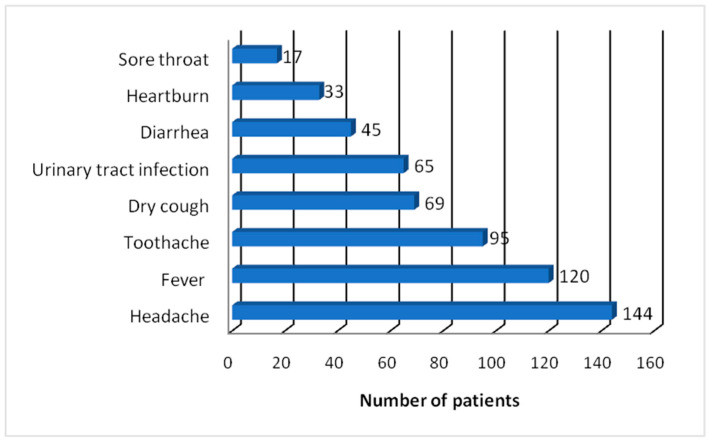
Symptoms and illnesses for self-medication practice by Tunisian CV patients.

When asked what medical reason led each respondent to practice self-medication, all mentioned headache (100%), 120 mentioned fever (83.33%), 95 mentioned toothache (65.97%), and 69 mentioned dry cough (47.92%) (Figure 2).

Urinary tract infection, self-reported as a reason for self-medication, was invoked by each patient, individually, with the help of urinary strips (diagnostic tests for urinary tract infection) either purchased from the pharmacy at the onset of urinary symptoms or pre-existing at the domicile of the surveyed persons. Other symptoms or illnesses included diarrhea, noted by 45 participants (31.25%), heartburn, noted by 33 participants (22.91%), and sore throat, noted by 17 participants (11.81%).

It must be noted that there were respondents who reported two or more medical reasons for self-medication (i.e., fever and dry cough, dry cough and headache, headache and heartburn, diarrhea and headache, sore throat and fever, etc.).

Based on the symptoms known from similar previous experiences, there were respondents who self-reported that they could self-diagnose themselves. They self-reported practicing self-medication to treat common conditions such as colds, sinusitis, tonsillitis, and urinary tract infections.

### 3.5. Pharmacological Type of Medicine Used for Self-Medication

The groups of medications commonly used by the 144 study participants who practiced self-medication are shown in Table 3.

The medicines self-reported by the study participants belonged to several pharmacological categories: analgesics, nonsteroidal anti-inflammatory drugs (NSAIDs), antimicrobials, cough suppressants, antacids, antidiarrheals, and birth control pills (Table 3). Paracetamol was the analgesic used by all respondents (CV patients) for the treatment of headache. More than half (59%) of the respondents used paracetamol alone, and the remaining (over 40%) used it in different combinations (paracetamol/codeine or paracetamol/caffeine). Paracetamol with caffeine was the preferred combination for almost one-third of the study participants.

Non-steroidal anti-inflammatory drugs (NSAIDs) were used by over 43% of respondents, with ibuprofen ranking first (18.06%) and diclofenac second (13.89%).

A significant proportion of patients (82; 56.94%) used antibiotics, such as amoxicillin in combination with clavulanic acid (24.31%), ciprofloxacin (17.36%), and gentamicin (15.28%). The patients self-reported using antibiotics to treat tonsillitis (17; 11.81%) and urinary tract infection (65; 45.14%). A total of 69 (47.92%) study participants used codeine to suppress dry coughs.

To reduce gastric acidity, 33 (22.92%) patients self-reported using the same antacid, an oral suspension containing sodium alginate and sodium bicarbonate.

Twenty of the female respondents used different birth control pills to prevent unwanted pregnancy.

### 3.6. Factors Associated with Self-Medication Practice

According to the descriptive statistical analysis presented in Table 1, CV Tunisian men are about 10 times more likely to practice self-medication compared to women. Additionally, the surveyed participants who had a moderate educational and low socio-economic level, who were smokers, and who lived at an urban residence were, respectively, 11 times, 13 times, 7 times, and 13 times more likely to practice self-medication compared to the other categories. The obtained wide confidence intervals (Table 1) can be attributed to the small size of the study sample.

Moreover, we were also interested in identifying whether there is a statistically significant correlation between socio-demographic variables or patients’ lifestyle habits and self-medication practice. To this end, we applied Fisher’s exact test to determine whether the frequency of self-medication practices correlated with the frequency of socio-demographic variables/lifestyle habits. The results revealed a statistically significant correlation between the practice of self-medication and male gender (*p* < 0.05), a moderate educational and socio-economic level (*p* < 0.05), urban residence (*p* < 0.01), and smoking (*p* < 0.05) (Table 4 and Table 5). More precisely, smoking men with a moderate educational and socio-economic level who lived in an urban environment were more inclined to practice self-medication compared to the study participants in the other categories.

## 4. Discussion

The aim of this study was to assess the prevalence and determinants of self-medication practices among CV patients from Béja, North West Tunisia, as the incidence of cardiovascular diseases continues to rise in Tunisia. To our knowledge, this is the first study to address this topic, aiming to better understand the extent of self-medication practices among this particular patient population. Simultaneously, the reasons determining or encouraging such a practice were also explored to obtain a socio-demographic profile of the CV patient in this Tunisian population segment predisposed to practicing self-medication.

Our cross-sectional study revealed a high prevalence of SMPs (96%) among participants who self-reported that they had used medicines at least once in the last 6 months without specialized medical/pharmaceutical consultations or recommendations.

Currently, Tunisia lacks studies demonstrating the current problems in the health system related to the practice of self-medication in the general population or in at-risk populations (children, the elderly, cardiovascular/diabetic patients, etc.). In this regard, the few studies carried out in the last 10 years in Tunisia have assessed the prevalence and determinants of self-medication, either in the general population [21,27] or in the pediatric population, with the latter being focused on self-medication with antibiotics [19,20].

On the other hand, recent studies have shown that worldwide (including developed and developing countries), SMPs among CV patients are present to a large extent; more precisely, SMPs were reported in over 70% of hypertensive patients [17], in 67% of CV patients in Canada (a study that evaluated both the use of OTC and herbal products) [28], and in 98.7% of patients enrolled in a primary cardiovascular healthcare program in Chile [18]. Considering this and the fact that SMPs in the general population of Tunisia reached a rate of 54.5% (according to a 2015 study) [21], our study findings are consistent with the trends observed in previous works utilizing surveys.

The differences between the results of various studies can be attributed to variations in the socio-demographic, economic, and geographical profiles of the respondents, as well as their educational, cultural, and religious differences. Moreover, an important role in the genesis of these differences in outcomes can be underlined by the following factors: different national health systems and their regulations, the existence or absence of the patients’ health insurance and its type, the period of the year in which the study was conducted and its duration, differences in the regulation and availability of non-prescription medicines in different countries, and the individual behavior of each patient. Notably, it should not be overlooked that there has been a marked increase in the prevalence of SMPs worldwide during the COVID-19 pandemic [29]. This context can also explain the high prevalence of SMPs among the CV patients, given that patients with CV disease are a high-risk population group for SARS-CoV-2 infection and severe forms of COVID-19 [30].

Other studies on SMPs by the general population reported different pharmacological classes, or the same, but with different frequencies of use, such as analgesics/NSAIDs [31,32,33], antibacterial drugs [31,34], and cough suppressants [32]; these studies are in accordance with our results.

Analgesics and NSAIDs were the most reported classes of medicines in SMP studies of hypertensive patients, followed by antitussives, antacids, and antidiarrheals [35,36,37].

Various personal reasons were attributed to SMPs by our respondents, such as the existence of a previous medical prescription (obtained for the treatment of similar symptoms or a similar previous medical condition) in 70.14% of cases, the lack of time to consult health professionals in 43.06%, and high costs of medical fees in 36.11%. The existence of a previous treatment was also reported by Scicluna and coworkers in their review [38] as the main reason for self-medication or as a source for SMP, while the other two personal reasons we identified (high cost of medical consultation and the lack of time to go to a medical consultation or saving time by avoiding it) are in accordance with the reasons reported by a similar Tunisian study performed in 2015 [21].

To alleviate or suppress various types of pain (acute or chronic and with various localizations), all patients resorted to the use of paracetamol (100%), alone or in combination with caffeine (31.25%), codeine (9.03%), or NSAIDs (43.75%). Paracetamol is the most widely recommended and used painkiller worldwide, on all continents, being mentioned in various studies in the general population and also by CV patients in Europe [39,40], Africa [31], America [28,41], Asia [42], and Australia [43]. The choice of this analgesic drug as the primary therapeutic option is in accordance with the guidelines of the new pharmacological strategies for the suppression of mild/moderate pain [44,45]. It was chosen by our respondents as the main treatment of various combinations of symptoms, of which the most common were the following: fever and headache, headache and toothache, headache, sore throat, and fever. Although most studies indicated that women use paracetamol, other painkillers, and NSAIDs [39,46] to suppress both acute and chronic pain more often than men, according to the results of our study, male respondents (64%) used them the most. However, our finding is consistent with the results of other studies about men self-medicating in general, performed in Turkey [47] and Saudi Arabia [48], showing that men are more likely to practice self-medication.

At present, the following is known about nonsteroidal anti-inflammatory drugs (NSAIDs) and CV events/risks: (i) NSAIDs increase the risk of CV events shortly after the onset of their use; (ii) their use by patients with CV disease increases the risk of a heart attack or stroke [49,50]; (iii) for diclofenac, the EMA’s Pharmacovigilance Risk Assessment Committee even issued a warning in 2013 about its CV consequences in the case of systemic administration of various pharmaceutical forms containing diclofenac [51]. However, in our study, the main NSAIDs consumed by our CV responders as self-medication were ibuprofen (26 patients, 18.06%) followed by diclofenac (20 patients, 13.89%). Ibuprofen is the main nonsteroidal anti-inflammatory molecule identified as being used as a self-medication for toothache. This result is in line with the recommendations of Tunisian dentists [52], according to whom, ibuprofen is their primary therapeutic option as an anti-inflammatory in oral infections of dental etiology (dental abscesses, postoperative pain in the dental sphere, etc.). These results are similar to those of other previous studies carried out on this particular population of hypertensive/CV patients in Trinidad [41], in the USA [37], and by our research group in Romania [53,54,55].

Because it is proven that a lot of socio-demographic factors influence SMP, we monitored the level of education and socio-economic status of the respondents, both reflected in the differences between patients’ health and the practices they use to regain it. The results of the present study revealed that the majority (80%) of the surveyed participants who practiced self-medication had a moderate (50%) or low (30%) educational level. The same trends were observed in terms of the socio-economic level of the majority of respondents (86.67%), namely moderate level (35.33%) or low level (51.34%), respectively. It is known that the education level of the patients is also reflected in their economic status. Under these conditions, one of the reasons that could support these results is that the group of participants with a moderate educational (secondary and post-secondary education, but not tertiary education) and low socio-economic level could not afford the cost of a visit to the doctor in addition to the cost of medication, which was also mentioned by 36.11% of patients as a personal reason for SMPs. According to the findings, it is possible that this recourse to SMP cannot be attributed to the isolation of the patient from health services (medical or pharmaceutical), as the present study was conducted in two pharmacies in urban areas and most patients (104; 70%) came from urban areas, with only 27 (18.75%) self-reporting that they were at a great distance from a physician.

Four of the analyzed common socio-demographic characteristics of the Tunisian CV patient evidenced by our study (man, under the age of 40, who lives alone and has a low income) were similar to those identified by Salem et al. in the general profile of the Tunisian patient who practices self-medication [21]. However, differences between the results of our and Salem et al.’s studies were found for other analyzed socio-demographic characteristics. For example, the present study revealed a moderate or low educational level in our CV patient group and SMPs among all age categories, compared to another Tunisian study that showed that a high educational level was present in 75% of participants. Additionally, among our self-medicated patients, 50.67% were inactive, coming from the categories of individuals without their own income (students, housewives, retired—who may be beneficiaries of old age, social, survivor, disability pension, etc.), compared to the other study where the share of inactive respondents was 32.6% [21].

It is worth mentioning that, in terms of socio-demographic characteristics of hypertensive patients practicing self-medication, the few previously published studies reported both that younger hypertensive patients were more likely than older patients to take over-the-counter (OTC) drugs for the treatment of minor symptoms or illnesses [35] and that men were more likely to self-medicate with complementary and alternative medicines [56,57].

The results of our study also showed that out of all the analyzed lifestyle habits of the respondents, cardiovascular smokers were more likely to self-medicate than non-smokers. This result supports the well-known relationship between patient exposure to tobacco and the onset of headaches [58]. This finding is similar to that previously reported by other studies that found that smokers had an increased and even double risk of suffering from headaches through drug abuse [59,60].

When analyzing the variables associated with self-medication, the conclusions of this study highlighted the presence of five variables in a statistically significant association with this practice: urban area (OR: 13.00; 95% CI: 1.4727–114.7515), male gender (OR: 9.6939; 95% CI: 1.1018–85.2907), moderate educational level (OR: 11.3846; 95% CI: 1.2163–106.5595), low socio-economic level (OR: 13.4118; 95% CI: 1.3134–136.9524), smoker status (OR: 6.7273; 95% CI: 1.1792–38.3785).

Another finding of the present study was the existence of an increased practice of self-medication with antibiotics (56.94%) and a careless attitude towards antibiotic resistance. Amoxicillin (in combination with clavulanic acid, a beta-lactamase inhibitor) was the antibiotic that, according to self-reports (in 24.31% of cases), was used most often, followed by ciprofloxacin (in 17.36% of cases) and gentamicin (in 15.28%) patients. These antibiotics were mainly used to treat sore throats—interpreted as tonsillitis (in 11.81% of cases)—and symptoms interpreted as urinary tract infection (in 45.14% of cases). SMP with antibiotics is well known in Tunisia, both in the general population and in the pediatric population. Our results are similar to other studies carried out in Tunisia, in which amoxicillin was the antibiotic reported and frequently used by patients, mainly for the treatment of upper respiratory tract infections in both the general population, with a prevalence of 20% in 2009 [38], and in pediatrics, with a prevalence increasing from 55% in 2010 [20] to 72.6% in 2021 [19]. Another reason for antibiotic self-medication was urinary tract infections treated with ciprofloxacin, gentamicin, or a combination of amoxicillin and clavulanic acid in 17.36%, 15.28%, and 12.5% of cases. This finding is consistent with a previous study in Tunisia [61] that suggested that fluoroquinolone, specifically ciprofloxacin, is the preferred molecule for the treatment of urinary tract infections in women and men.

Self-diagnosed infections, choosing antibiotics based on the criterion “I have an old prescription”, and incorrect dosing or treatment duration contribute to irrational and abusive antibiotic use. These factors favor and increase the development of antimicrobial resistance and treatment failure.

According to our findings, a significant proportion of respondents (47.92%) used drugs such as codeine (tablets or syrups) in self-medication for the purpose of improving symptoms of the upper respiratory tract (i.e., dry cough). In addition to these, a further 9.03% of survey participants self-reported the self-use of a combination of codeine and paracetamol for a stronger analgesic effect. Codeine is known to be used in combination with paracetamol to increase the analgesic effect and can be purchased from community pharmacies with or without a prescription, depending on the country. In Tunisia, this combination is available in two formulations: 300/600 mg paracetamol + 25/50 mg codeine per tablet. It is important to note that the repeated use or misuse of this combination has been found to lead to medication-overuse headache [62]. Consuming codeine alone or in various combinations without a medical recommendation regarding the correct dose regimen and duration of use increases the risk of its misuse, as well as the development of side effects, tolerance, and dependence [63].

It is important to note that antibiotics and all medicines containing codeine (for cough suppression but also as an analgesic in combination with paracetamol) are available in Tunisia only on prescription.

## 5. Recommendations for Healthcare Professionals

Our study revealed that the practice of self-medication among Tunisian cardiovascular patients is an important public health issue and should be closely monitored.

The results of this study cannot be generalized to the entire population of cardiovascular patients in Tunisia. However, the data provided underscore that healthcare professionals need to be aware of the existence of this practice among patients with cardiovascular disease. This awareness creates opportunities for discussions with patients about the risks of using of medications without medical advice. Encouraging patients to report such practices, namely the medicines they use, is crucial. Physicians and pharmacists should be aware of the consequences of SMPs, including increased risks of potential side effects, drug–drug interactions, therapeutic failure or worsening of cardiovascular disease, drug dependence, etc., particularly given the complex and multiple-drug regimens of CV patients.

## 6. Strengths of the Current Study

As far as we know, this is the first article addressing SMPs among Tunisian cardiovascular patients. Moreover, it is one of few works to assess the practice of self-medication among this highly vulnerable and at-risk population of patients. Given that cardiovascular diseases are the leading cause of death worldwide, irrational drug use in the practice of self-medication can contribute to a deterioration of quality of life, increase health insurance costs, and, last but not least, increase the overall morbidity and mortality.

## 7. Study Limitations

This study has several limitations, including the following: (1) the small sample size of the surveyed CV patients; (2) the small number of registered community pharmacies; (3) the short duration of the study and possibly even the period of the year chosen for its development; (4) using a cross-sectional study design, meaning that causal relationships between the various variables could not be analyzed and established; and (5) reliance solely on self-reporting data from patients, which may lead to under/overreporting of information.

## 8. Conclusions

In this study, a high prevalence of self-medication practices in Tunisian patients with cardiovascular disease was reported. The common reasons for this practice were the existence of an old prescription and the saving of time and/or money. Headache, fever, toothache, cough, and urinary tract infection were the main conditions of the participants, leading them to resort to self-medication. The most frequently self-administered drugs were paracetamol, antibiotics, codeine (as a cough suppressant), and NSAIDs. Factors significantly associated with the practice of self-medication were male gender, smoking, urban area of residence, living alone, young or elderly, self-employed or retired, a moderate level of education, and low economic status.

The widespread use of over-the-counter drugs by cardiovascular patients, particularly those not prescribed/recommended by a therapist (physician/pharmacist), increases the risk of drug–drug/pathology interactions, therapeutic failure, aggravation of these diseases, and frequent adverse events. From the point of view of medical and pharmaceutical professionals, individuals in at-risk population groups, such as the cardiovascular patients included in this study, should be prioritized for participation in public health education programs that aim to improve the quality of their behavior towards self-medication and, implicitly, their quality of life. Understanding the reasons behind the self-medication practices of cardiovascular patients, as well as knowing the socio-demographic profile of patients who frequently engage in these practices and the non-prescription drugs they use, is important in successfully managing cardiovascular disease, not just in Tunisia but around the world.

## Figures and Tables

**Table 1:** The prevalence (*n*, %) of self-medication practices associated with the socio-demographic characteristics and lifestyle habits of the study participants; odds ratios with 95% confidence intervals (OR, 95% CI) and *p*-values. *—statistical significance.

Variables	Category	SMP Yes (*n* = 144; 96%)	SMP No (*n* = 6; 4%)	OR (95% CI) *p*-Value
Socio-demographic characteristics				
Gender	Female	49	5	1 (reference)
	Male	95	1	9.69 (1.10 to 85.29) *p* = 0.04 *
Age group	20–39 years	49	1	1 (reference)
	40–59 years	28	2	0.29 (0.02 to 3.29) *p* = 0.31
	60–69 years	40	2	0.41 (0.04 to 4.67) *p* = 0.47
	70–79 years	27	1	0.55 (0.03 to 9.16) *p* = 0.68
Marital status	married	18	2	1 (reference)
	divorced	31	1	3.44 (0.29 to 40.71) *p* = 0.33
	widowed	67	1	7.44 (0.64 to 86.81) *p* = 0.11
	Single	28	2	1.56 (0.20 to 12.05) *p* = 0.67
Level of education	Low	44	1	6.77 (0.72 to 63.86) *p* = 0.09
	Moderate	74	1	11.38 (1.22 to 106.56) *p* = 0.03 *
	High	26	4	1 (reference)
Residence	Urban	104	1	13.00 (1.47 to 114.75) *p* = 0.02 *
	Rural	40	5	1 (reference)
Employment status	Retired	45	1	2.27 (0.26 to 20.02) *p* = 0.46
	Self-employed	35	1	1.61 (0.18 to 14.21) *p* = 0.67
	Housewife	24	1	1.00 (0.11 to 8.95) *p* = 1.00
	Employed	14	1	0.54 (0.06 to 4.94) *p* = 0.58
	Student	5	-	-
	Trader	13	1	0.50 (0.05 to 4.58) *p* = 0.54
	Farmer	9	1	0.33 (0.04 to 3.16) *p* = 0.34
Socio-economic level	Lower	76	1	13.41 (1.31 to 136.95) *p* = 0.03 *
	Moderate	51	2	4.50 (0.69 to 29.24) *p* = 0.12
	High	17	3	1 (reference)
Patient’s lifestyle habits				
Smoker status	smoking	111	2	6.73 (1.18 to 38.38) *p* = 0.03 *
	non-smoker	33	4	1 (reference)
Alcohol intake	Yes	53	2	1.16 (0.21 to 6.58) *p* = 0.86
	No	91	4	1 (reference)
Dietary practices	Yes	85	1	7.20 (0.82 to 63.25) *p* = 0.07
	No	59	5	1 (reference)
Physical activity	Yes	60	2	1.43 (0.25 to 8.05) *p* = 0.69
	No	84	4	1 (reference)

**Table 2:** Distribution of CV patients (*n*, %) according to self-reported personal reasons for self-medication.

Items	Frequency of Subjects Surveyed	Percentage
I have an old prescription	101	70.14%
I save time	62	43.06%
Medical fees are high	52	36.11%
The physician is busy with a lot of patients	32	22.22%
The physician/clinic is too far from me	27	18.75%

Note: The number of respondents who practiced self-medication (144) was used as the denominator in the computation of percentages; the sum of percentages exceeds 100% as many respondents gave two or more answers to this question.

**Table 3:** Self-reported medicines used by the study participants according to the pharmacological class.

Pharmacological Classes of Drugs Used as Self-Medication	Frequency	Percentage
Analgesics	144	100%
Paracetamol	86	59.72%
Paracetamol + caffeine	45	31.25%
Paracetamol + codeine	13	9.03%
Non-steroidal anti-inflammatory drugs (NSAIDs)	63	43.75%
Ibuprofen	26	18.06%
Diclofenac	20	13.89%
Acetylsalicylic acid	17	11.81%
Antibiotics	82	56.94%
Ciprofloxacin	25	17.36%
Amoxicillin + clavulanic acid	35	24.31%
Gentamicin	22	15.28%
Cough suppressants	69	47.92%
Codeine		
Antacids	33	22.92%
Sodium alginate + sodium bicarbonate		
Antidiarrheals	45	31.25%
Birth control pills	20	13.89%

**Table 4:** Relationship between patient’s socio-demographic variables and the presence of self-medication practices in the study sample.

Variables	Category	SMP Yes (144)	SMP No (6)	*p*-Value ^a^
Gender	Female	49	5	0.023 *
	Male	95	1	
Age group	Under 60 years	77	3	1
	Over 60 years	67	3	
Marital status	Married	18	2	0.1825
	^b^ Unmarried	126	4	
Education	<High level	118	2	0.0149 *
	High level	26	4	
Residence	Urban	104	1	0.0095 *
	Rural	40	5	
Employment status	Employed	14	1	0.4746
	Unemployed	130	5	
Socio-economic status	<High level	127	3	0.0315 *
	High level	17	3	

^a^ *p*-values are based on Fisher’s exact test; * statistical significance at *p* < 0.05; ^b^ divorced, widowed, or living alone patients.

**Table 5:** Relationship between patient lifestyle habits and the presence of self-medication practices in the study sample.

Variables	Category	SMP Yes (144)	SMP No (6)	*p*-Value ^a^
Smoker status	Smoking	111	2	0.0328 *
	Non-smoker	33	4	
Alcohol intake	Yes	53	2	1
	No	91	4	
Dietary practices	Yes	85	1	0.084
	No	59	5	
Physical activity	Yes	60	2	1
	No	84	4	

^a^ *p*-values are based on Fisher’s exact test; * statistical significance at *p* < 0.05.

## Data Availability

All data available are reported in the article.

## Supplementary Materials

The following are available online at https://www.mdpi.com/article/10.3390/pharmacy12020068/s1, Form S1: Pharmacy survey form.
