# Supplementary material for: Prevalence and Determinants of Self-Medication Practices among Cardiovascular Patients from Béja, North West Tunisia: A Community-Pharmacy-Based Survey
Source: Pharmacy (Basel). 2024 Apr 12;12(2):68. doi: 10.3390/pharmacy12020068 (PMC11054241; doi:10.3390/pharmacy12020068)
Supplement: Supplementary file 1 [file pharmacy-12-00068-s001.zip › pharmacy-2903437-supplementary-1.pdf]

Form S1

Pharmacy survey form:

Screening date : _ _/_ _/_ _<br>No. Subject: ………………..	Age : ....
Gender : Male Female	Language: ……………...

Please complete the following form :

Questionnaire to assess self-medication practices in cardiovascular patients compared to the general population in a community pharmacy in Tunisia

Note: The private information provided in this questionnaire is used to draft a brief and we are grateful for all survey responses. All private and personal data is collected anonymously and will be presented in statistical form. Thank you for your understanding.

I. GENERAL INFORMATION:

1. **Marital status :**
□ Single □ Married □ Widow(er) □ Divorced □ IDK/IDWA

2. **Level of education:**
□ Primary □ Secondary □ Higher □Unschooled □ IDK/IDWA

3. **Place of residence:** □ Urban environment □ Rural environment □IDK/IDWA

4. **Socio-economic level :**
□ Low □ Medium □ High □ IDK/IDWA

5. **Employment situation:**
□ employee □ retailer □ farmer
□ independent profession □ unemployed □ student
□ pensioner □ other (housewife) □ IDK/IDWA

II. PERSONAL HABITS :

6. **Do you smoke ?**
□ current smoker □ non-smoker □ former smoker
If yes, since when ?..........................and how many cigarettes a day ? ....

7. **Do you drink alcohol ?** □ Yes □ No

- If yes, since when ?......................... and how many times a week ? ....

III. PERSONAL PATHOLOGICAL ANTECEDENTS:

8. Do you suffer from cardiovascular diseases?

- □ Yes □ No □ IDK/IDWA
- **If yes, which one? ....**

HTA □ Coronary heart disease : (MI □ , angina □) AVC □ Atherosclerosis □

Lower limb arteriopathy □ Heart failure □

Valvulopathy □ Heart rhythm disorders □ Other ....

9. Since when … ?

□ one month □ less than 3 months □ between 3 months and 1 year

□ more than 1 year □ more than 5 years □ IDK/IDWA

10. Please indicate what other health problems you have :

- Bronchial asthma □ BPOC □ Peptic ulcer disease
- Dyslipidemia □ Hypertriglyceridemia
- Hypercholesterolemia □ Diabetes mellitus type I □ Diabetes mellitus type II
- Hyperthyroidism □ Obesity □ Overweight
- Anxiety □ Depression □ Menopause
- No associated pathology

ϒ Other pathology...□ IDK/IDWA

IV. MEDICATION MANAGEMENT :

Please allow me to define what self-medication is:

„ Self-medication is the use of medicines (by the consumer) that are not prescribed by the doctor."

11. Have you self-medicated in the past six months?

□ Yes □ No □ IDK/IDWA

12. What was the reason for your self-medication?

(You can select multiple choices by ticking (×) the appropriate boxes below)

□ Doctor/clinic far from home □ Time-saving

□ High Doctor's fees □ I have an old prescription

□ The doctor is busy with a lot of patients

□ I have medication from family members

□ No trust in the doctor
□ Prefers pharmacist's advice
□ Other. Explain ……………… □ IDK/IDWA

13. **For what diseases/symptoms and signs have you self-medicated for in the past six months?** (You can select multiple choices by ticking (×) in the appropriate boxes below)

headache	migraine	sore throat	toothache	earache
fever	runny nose	cough	nausea and vomiting	gastric burns
acidity	stomach pain	diarrhea	constipation	haemorrhoids
insomnia	joint pain	muscle pain	slimming	menstrual pain
menopause	conjunctivitis	dry eyes	pregnancy prevention	fatigue
hypertension	bronchial asthma	diabetes	impotence	urinary tract infection
difficulty in urinating	genital infection	varicose veins	hives	boil/abscess

Other reasons ……………………………………………….

14. **What are your considerations when choosing a drug for self-medication?**
□ Price □ Pharmaceutical company □ Type of medication
What type of medication do you choose?
□ Naturist □ Allopathic
□ Homeopathic □ Dietary supplement
□ Other. Explain ……………………………..

15. **Where do you get your medication for self-medication?**
□ Pharmacy □ Online shopping
□ Primary Health Care Centre □ Medical Representatives
□ Supermarket □ Station service
□ Family/Friends □ IDK/IDWA
□ Other ………………………………….

16. **Do you check prescription information before self-medicating ?**
□ Yes, always □ No, never □ Yes, sometimes

17. **How do you take these products?**
□ on your own initiative □ on referral from a friend/family
□ by renewing a prescription □ according to the TV/Press commercial

18. **Please mention the product you bought?** (Fulfill its trade name)

………………………………………………………………………………….

19. How long have you been taking this preparation as self-medication?

□ this is the first time □ less than 3 months □ between 3 months and 1 year
□ more than 1 year □ more than 5 years □ IDK/IDWA

20. What results have you obtained after using these preparations?

□ complete satisfaction □ low satisfaction
□ no results □ side effects

21. Have you ever experienced adverse effects taking self-medication?

□ Non □ Yes

- **If yes, explain ....**

22. What did you do about the adverse effects you experienced ?(the adverse effect(s) refers to question 21)

□ Going to the private doctor □ Go to the pharmacist
□ Going to the hospital □ Stop taking medication
□ Other. Explain..□ Reduce the dose

23. When did you decide to stop consuming these medicines?

□ when I finished the box □ in the event of no effect
□ if symptoms worsen □ IDK/IDWA
□ others. Please explain ………………………

24. Are you taking medication for your cardiovascular condition ?

- □ Yes □ No □ IDK/IDWA
- If yes, which one? ....
- For which type of pathology? ....

25. Are you currently taking any other medication ?

- □ Yes □ No □ IDK/IDWA
- If yes, which one? ....
- For which type of pathology? ....

Thank you for your time!

□ **IDK/IDWA** = don't know / don't want to answer
